# Supplementary material for: Complexome profiling of the Chlamydomonas psb28 mutant reveals TEF5 as an early PSII assembly factor
Source: Plant Cell. 2025 Mar 17;37(6):koaf055. doi: 10.1093/plcell/koaf055 (PMC12164590; doi:10.1093/plcell/koaf055)
Supplement: koaf055_Supplementary_Data [file koaf055_supplementary_data.zip › psb28_interactive.zip!/plots/intensity/qualityPlots/peptides/_Cre06.g259450.t1.2.html]

### Cre06.g259450.t1.2

Trivial name:   
SeqCoverage [median]: 37.20  
Mass: 23.79 kDa  
  
 

| Sequence | MissedCleavage | Mass [Da] | wtIntensity | muIntensity | Score |
| --- | --- | --- | --- | --- | --- |
| AGGAGAADAVYAALGR | 0 | 1389.70 | 4827.03 | 18708.59 | 232.00 |
| AVAEAAPVAVAAVTGAADKEETLGEMYGGK | 1 | 2875.43 | 0.00 | 6040.58 | 76.24 |
| EIEALAATPEDSEER | 0 | 1658.76 | 0.00 | 931.94 | 169.65 |
| EIEALAATPEDSEERFR | 1 | 1961.93 | 0.00 | 5434.35 | 126.63 |
| LPAPLLDEAQAFR | 0 | 1439.78 | 469.22 | 0.00 | 68.89 |
| VLTISGR | 0 | 744.45 | 1856.77 | 11530.36 | 125.08 |
|  |  |  | 7153.0 | 42645.8 |  |
